# Supplementary material for: The effect of respiratory gases and incubation temperature on early stage embryonic development in sea turtles
Source: PLoS One. 2020 Dec 2;15(12):e0233580. doi: 10.1371/journal.pone.0233580 (PMC7710074; doi:10.1371/journal.pone.0233580)
Supplement: Attachment — Submitted filename: Response to reviewers.docx [file pone.0233580.s003.docx]

PONE-D-20-13033
The effect of respiratory gases and incubation temperature on early stage embryonic development in sea turtles
PLOS ONE

Dear Editors,

Please thank the reviewers for their comments and queries which we have addressed in our revised manuscript. We believe the revised manuscript is an improvement on the original, and hope that you now find it suitable for publication in PLOS ONE. Below we show how we have addressed each of the reviewer’s comments. Our responses are in red font.

Journal Requirements:

 Reviewer's Responses to Questions

**Comments to the Author**

1. Is the manuscript technically sound, and do the data support the conclusions?

Reviewer #1: Yes

Reviewer #2: Partly The issues raised by this reviewer have been rectified

2. Has the statistical analysis been performed appropriately and rigorously?

 Reviewer #1: Yes

Reviewer #2: No The issues raised by this reviewer have been rectified

3. Have the authors made all data underlying the findings in their manuscript fully available?

The [PLOS Data policy](http://www.plosone.org/static/policies.action#sharing) requires authors to make all data underlying the findings described in their manuscript fully available without restriction, with rare exception (please refer to the Data Availability Statement in the manuscript PDF file). The data should be provided as part of the manuscript or its supporting information, or deposited to a public repository. For example, in addition to summary statistics, the data points behind means, medians and variance measures should be available. If there are restrictions on publicly sharing data—e.g. participant privacy or use of data from a third party—those must be specified.

 Reviewer #1: No Data is available by attached supporting information

Reviewer #2: Yes

4. Is the manuscript presented in an intelligible fashion and written in standard English?

Reviewer #1: Yes

Reviewer #2: Yes

5. Review Comments to the Author

Reviewer #1: General points:

The manuscript presents a sound study that answers an important biological question with real conservation consequences for threatened sea turtle populations. I congratulate the authors on designing and executing a valuable study to elucidate the causes of abnormally high early-stage embryonic death at high-density sea turtle nesting beaches. We thank the reviewer for these comments.

My main criticism with this manuscript is that, for the two clutches where data was presented for stage of embryonic mortality, the temperature treatments were applied over the entire incubation period and therefore does not reflect a "short-term" early incubation effect of a nearby mature clutch incubating. In the clutches where high temperatures were only applied for the initial period of incubation the authors have not presented the data for stage of embryonic development at which the embryos died. I suggest that the authors either revise the manuscript to include the stage of death data for the unhatched eggs from clutches that were reburied in artificial nests. If these data do not exist, the authors need to address this limitation and I suggest they include a caveat in the discussion to highlight the limitation that embryonic death cannot be conclusively attributed to "short-term early-stage" high incubation temperature. This is the only major change I recommend making to the manuscript, the rest of the points I raise should be easy to address. Both reviewers comment on this point. To simplify matters we now only confine the manuscript to the first week of incubation and exclude the data on development and mortality later in incubation.

What about the impact of extended pre-ovipositional embryonic arrest on embryos ability to survive high nest temperatures and low oxygen partial pressure? At high-density nesting beaches, such as Raine Island and Ostional, it could be likely that there are higher rates of failed nesting attempts due to disturbance from other females. This would mean a higher proportion of females return to nest over subsequent days to attempt oviposition when compared with those nesting at low-density nesting beaches. Inherently, this would mean that embryos are maintained in embryonic arrest for longer periods of time. Previous experiments have found that sea turtle embryos have limited tolerance to remain in embryonic arrest (Rings et al. 2015 Phys. Bio. Zool. and Williamson et al. 2019 Sci. Rep.) and in one population of leatherback turtles longer interesting intervals result in higher proportions of early-stage embryonic mortality (Rafferty et al. 2011 PLoS One). If embryos are potentially compromised by extended arrest they may be more susceptible to higher incubation temperatures and lower oxygen availability when they do recommence development after the arrest. It would be good for the authors to include some acknowledgement of this possibility in the discussion. This interesting possibility is now included in the discussion by the addition of the paragraph: “We collected the eggs for our experiments from low density nesting populations in which we knew by checking flipper tag numbers that the females laid their clutch on their first laying attempt. Previous reports [Rafferty et al. 2011, Rings et al. 2015, Williamson et al. 2019] indicate that retention of eggs for several days within the female reproductive tract due to multiple failed nesting attempts which frequently occurs at high nest density beaches, causes a decrease in egg viability. Hence, it is possible that at high nest density beaches a combination of prolonged egg retention and exposure of eggs to low oxygen, high carbon dioxide partial pressures and high nest temperatures result in high early stage embryo mortality.

Specific points:

Page 1, Line 14: Change to "high death rate" or "high mortality rate". Done.

Page 1, Line 22: Change "sea turtle nest" to "sea turtle nests". Done.

Pages 1 - 2, Lines 23 - 24: Change "did not influence embryo mortality of early stage embryos" to "did not influence the mortality rate of early stage embryos". Done.

Page 2, Line 25: I am unsure what the current word count for the abstract is, but I suggest including a final sentence summarizing the importance and implications of the findings. We have now added a concluding sentence to the abstract: “Our findings indicate that low oxygen and high carbon dioxide partial pressures are not the cause of high early stage embryo mortality observed at high density sea turtle nesting beaches.”

Page 2 Line 34: Delete "of" from "increases of lizard" Done.

Page 2, Lines 36-37: Provide a reference for this sentence. Perhaps one of the Ackerman papers on the topic. I suggest also breaking this paragraph into two separate paragraphs. One on the information about developmental arrest and the other on the information about varying gas concentrations and temperature throughout incubation in the nest. A reference (Ackerman 1997) is now provide and this paragraph is now broken into two separate paragraphs.

Page 2, Line 39: Change "stalls" to "arrests". Done

Page 2, Lines 39 - 40: Embryos do not break developmental arrest immediately after oviposition. The study that the authors cite for this sentence suggests that embryos break the arrest after at least 12 hours of exposure to higher oxygen partial pressures. Please change this sentence to reflect this difference. This sentence has been re-written: “Once the eggs are laid, they are exposed to higher oxygen partial pressures so that the oxygen limitation is lifted and the embryos break developmental arrest about 12 hours after laying and recommence development [4].”

Page 2, Line 42: Change "development arrest" to "developmental arrest". Done.

Page 3, Line 53: Change "it released carbon dioxide" to "it releases carbon dioxide". Done.

Page 3, Line 57: Change “aggregations such as” to “aggregation beaches, such as”. Done.

Page 3, Line 58: Arribadas is plural in Spanish. Change "nesting in arribadas aggregations" to "nesting in arribadas" or "nesting in arribada aggregations" Done.

Page 3, Line 68: Again, this paragraph is very long. Suggestion to split into two paragraphs after this sentence. Done.

Page 3, Lines 70-72: Provide appropriate references for this section of the sentence. Possibly Nicki Mitchell et al papers on heat-shock proteins in sea turtle embryos e.g. Bentley et al. 2015 Mol. Ecol.; or Tedeschi et al 2015 or 2016. Tedeschi et al. 2015 is now cited.

Page 5, Line 111: Please provide more information on the type of containers used for the application of the gas treatments. This information is now included: “We then placed eggs into a gas tight plastic container (Sistema Klip, 4L, 20cm x 20cm x 10 cm) labelled with its temperature and respiratory gas mixture treatment.”

Page 5, Lines 113 - 114: Unless I have missed it, there is no explanation provided for the use of Raine Island sand during incubation. Please explain the reasoning for this. We have added an explanatory sentence: “We used this sand because Raine Island it is host to high-density and many nests experience high early stage embryo mortality.”

Page 6, Line 131: Provide reference to the staging criteria that you staged embryos with. I assume Miller's 1985 staging guide? This reference was mis-labelled here, be now cite the correct reference (Miller et al. 2017).

Page 7, Line 159: Please provide clarification whether these eggs were maintained at their assigned temperature treatment or if all eggs were then incubated at a similar temperature. This part of the study has now been deleted from the manuscript.

Page 7, Line 160: Please explain a little more about what happened to these eggs. Were they opened and staged to assess viability? Were the eggs left to incubate until hatching in the incubators? This part of the study has now been deleted from the manuscript.

Page 8, Lines 188-191: I assume you excavated and staged the unhatched eggs from the clutches that were reburied in artificial nests as well. You should provide a summary of these data also. Or explain why this was not done. If the primary concern of this study is early stage death I think it is quite important to present the data on which stage of development these embryos died at. This part of the study has now been deleted from the manuscript.

Page 9, Line 198: This is an important finding and indicates that growth of the white spot is actively regulated by the embryo rather than passively by desiccation or other environmental influences. Thanks for pointing this out.

Page 10, Line 228: Change "logger turtle" to "loggerhead turtle" Done.

Page 10, Lines 235-237: It is important the authors have acknowledged this potential limitation in assessing the difference in growth rates between the two species given that they applied different methodologies to the eggs dependent on species. Thanks for this comment.

Page 12, line 274: Change "early stage sea turtle embryos development rate" to "the development rate of early stage sea turtle embryos". Done.

Page 19, Lines 426 – 427 and Page 20, Lines 430 - 431: Table 1 & 2 Captions are nonsensical to me. The experimental treatments cannot indicate survival in themselves. I suggest changing "turtle eggs indicating survival" to "turtle eggs and embryo survival" or something similar. These captions have been changed as suggested; Table 1: “Experimental treatments used to incubate loggerhead turtle eggs and the absolute number as well as the proportion of embryos alive at day 7 after exposure to the incubation treatment.” Table 2: “Experimental treatments used to incubate green turtle eggs and the absolute number as well as the proportion of embryos alive at day 7 after exposure to the incubation treatment.”

Reviewer #2: Generally, I found the study by Booth et al. interesting and relevant from a conservation perspective and the collected data is suitable to answer the main study question. The manuscript itself is concise, well-written with appropriate referencing. However, my major concern is that the experimental design is unclear and does not always seems to address the main study question, which could be amended by a careful revision of the M&M section. In addition the authors draw conclusions that are not supported by their data or their experimental approach:

i) by comparing the two study species, which is not supported by statistics or a comparable handling of the embryos: This comparison has been deleted from the revised manuscript.
ii) ii) by concluding that development is affected by hypoxia, which was not tested independently from hypercapnia: This point has been rectified in the revised manuscript.
iii) and iii) by confounding the results from a short-term exposure in a common garden experiment with the results from a long term survival trial (where embryos were pre-exposed to different gas tensions, but then kept in ambient air): Results and discussion of the longer duration experiment have been deleted from the revised manuscript.
iv) I think all of these concerns can be addressed, but will require re-analyzing some of the data, changing the presentation of the results and re-writing large parts of the discussion to adhere more closely to the study question and the data at hand. Therefore, I cannot recommend the manuscript for publication in PlosOne in its current form and in the following I list 5 major concerns that I would like the authors to address. In addition, I have listed a number of minor comments that will hopefully help with the revision of this manuscript. We have re-written major sections of the manuscript to address all of these reviewer’s concerns.

1. The discussion is well-written and meaningful as far as it pertains to the main research question. But to a large extent the authors take the discussion beyond what can be concluded from their data to an extent that is unacceptable for publication. I recommend the authors adhere closely to the main research question and avoid over-interpreting their data. For instance , the authors conclude that one species is more susceptible to hypoxia than the other, based on a metric of developmental rate (L227-245), but this isn’t supported by any statistical analysis, the embryos were not treated in the same way prior to experiments and generally potential interspecific differences in developmental rate are not considered. Furthermore, the authors discuss potential effects of hypoxia on development, but hypoxia was always tested in combination with hypercarbia and therefore such mechanistic distinctions cannot be made (L238-245). I urge the authors to fundamentally revise the interpretation of their data and pay close attention to the limitations in their experimental design. These issues have now been deleted from the discussion in the revised manuscript.

2. I think the experimental design is appropriate to answer the main study question on whether early embryonic development/survival is affected by the conditions (gas and temperature) created by nearby clutches on crowded beaches. This was assessed in a first experiment over 7 days where embryos were exposed to different combinations of gas tensions and temperature and development was monitored. This experiment answers the main study questions and there were no effects on early embryo survival, but development was delayed. After this, things get more confusing and the further experiments are not in line with this initial hypothesis. The second experiment looked at long-term survival of the embryos at different temperatures, but at ambient gas tension (and using the embryos that had been previously exposed to different gas tensions). Here the authors report significant differences in survival, but it’s unclear whether this longer time-frame addresses the initial question about “early embryo mortality” (a term that should be clearly defined early in the paper) and fails to address the actual conditions on crowded beaches that always include altered gas tensions. The presentation and discussion of the results intermixes the findings from these two experiments and it is often unclear which condition is being discussed. I suggest the authors outline more clearly the justification for this second experiment and how it fits within the main study objective. One option would be to re-analyze the data and test whether an early exposure of the embryos to altered gas tensions had an effect on long-term survival even at atmospheric gas tensions (currently the results from all gas-tension pre-exposures are pooled across the temperature treatments). The findings of this analysis would be interesting regardless of the outcome and could tie the two experiments together to test a common hypothesis. These issues have been fixed in the revised manuscript by deleting the data and discussion for the second long-term experiment.

3. In general, the way the authors have described the experimental design of their study is convoluted and even after reading the manuscript again it is not entirely clear to me why certain conditions were tested. I recommend that the authors include a dedicated section in the M&M on experimental design, where they lay out clearly what the experimental treatments were, when each treatment was sampled and how the treatments were replicated (the authors use eggs as N whereas really each clutch should be an experimental unit, where N=4). I also recommend dividing the experiments into clearly defined stages (phases or series). The first from 0-36 h after which measurements were taken, the second 36 h- 7d which tested the effects of gas tensions and temperature on development and finally a long-term exposure 7-46 d in which only survival was assessed. The methods section has been comprehensively revised, and the confusion generated by the second longer-term experiment eliminated by deleting this experiment from the revised manuscript.

4. I recommend the authors revisit the presentation of their data. Figures 2 and 3 show both species in one graph, whereas gas tensions were pooled for each temperature. I don’t think this is a valid presentation of the data as there were significant differences between the gas tensions and therefore, they shouldn’t be pooled in the first place. In addition, there was a significant interaction effect between gas tensions and temperature and therefore all combinations of temp*gas need to be shown separately. I recommend showing the p-values of main effects and interactions in the figure as well, as this reflects the outcome of the statistical analysis and Tables 3 and 4 could be omitted. And finally, I suggest presenting one figure per species, as no statistical comparisons are made between the two species.
5. In one of the treatments the pre-mixed gases ran out and the authors chose to convert this treatment into a high-temperature exposure (as far as I understood this section) where embryos were exposed to 34°C instead of 33°C. It is not clear why this additional increase in 1°C was chosen and, without any context, it is surprising that it had large effects on animal survival. I recommend the authors justify their approach more clearly. Since the other gas tensions were pooled by temperature this 34°C exposure is the only treatment that received ambient air throughout development (correct?) and therefore is not necessarily comparable to the other treatments. One way to avoid this confounding effect of pre-exposure to different gas tensions would be to compare the 34°C exposure only to the other treatments that were exposed to ambient air throughout (controls). I suspect the results would still hold up and this would make for a more meaningful comparison (if the results don’t hold up this is reason for concern in itself). These suggestions have been taken on-board in the revised manuscript. Figure 1 is deleted as are all results and discussion relating to the longer-term experiment. Only one figure is now included in the revised manuscript and it has been re-drawn to conform with the reviewer’s requests.

Specific comments:
Abstract
L13-14 Here and in the introduction, please define the term “early stage embryo death”. This is especially important since the study assessed embryo development over two distinct time-frames and throughout the manuscript it is unclear whether both parts of the study address the issue of the early mortality of embryos. We now define and early stage embryo”: “Although these altered environmental conditions are well tolerated by mature embryos, early stage embryos, i.e. embryos in eggs that have only been incubating for a week, may not be as tolerant leading to an increase in their mortality.” The part of the study using the second timeframe has been deleted from the revised manuscript so this confusion is now avoided.

I find the last sentence of the abstract a bit cryptic and, based on this, I was unsure whether there was a clear effect of temperature on embryo mortality. Consider rephrasing this sentence for clarity. Also, I’d like to see a concluding statement in the abstract that is based on the current findings. What does it all mean for wild turtles that nest in crowded beaches? The end of the abstract has been re-written to address these issues: “Our findings indicate that low oxygen and high carbon dioxide partial pressures are not the cause of high early stage embryo mortality observed at high density sea turtle nesting beaches, but there is some evidence to suggest high incubation temperatures, particularly above 34oC are harmful. Any management strategies that can increase the spacing between nests is likely to increase hatching success at high density nesting beaches.”

Introduction
L34 delete “of”. Also, it’s worthwhile mentioning that these were embryos of live-bearing lizards and I would note the level of hypoxia and the temperatures, as well. This adjustment has been made in the revised manuscript: “For example, lethal temperature decreased from 44^o^C to 40^o^C as oxygen decreased from 21% to 10% in a viviparous lizard.”

L45 replace that” with “those” Done.

L49 I would not use the term peak metabolism, because it implies that metabolism would decrease again throughout embryonic development. Presumably this refers to the last stage in development before hatch. We left this unchanged because sea turtle embryonic reaches is maximum a few days before hatching, and metabolic rate actually decreases from this peak before hatching occurs.

L53 “releases” Done.

L51-54 I agree that hypoxia and hypercarbia typically go hand-in-hand in natural settings, but the effects on the organisms differ greatly between these two conditions. Overall, I found the paper focused only on O2 whereas the experimental treatments manipulated both gases. I suggest the authors introduce briefly the effects of hypercapnia on embryonic development as well; many good physiological studies have recently dealt with this. At the reviewer’s suggestion, the section of the discussion discussing oxygen limitation has been deleted, so it would be inappropriate to include discussion on carbon dioxide effects as the reviewer points out our data cannot distinguish between possible oxygen and carbon dioxide effects.

L54-54 What are typical temperatures in the sand? Provide some context so the reader knows if we’re talking about a 30% (sand temp ~10°C) increase in temperature or a 10% increase (sand temp ~30°C). Done: “The combined metabolism of embryos also generates considerable heat which causes the nest temperature to rise between 2-5^o^C above the surrounding sand temperature (typically 28 – 32^o^C) during the latter part of incubation [8,9].”

L56 final stages of incubation Done.

L57-60 I found this sentence rather vague, but it is a critical justification for conducting this study. Please state specifically whether within one species crowded nesting beached have higher embryo mortality rates compered to empty beaches. Be specific and mention how large of an effect this is for each species. As written, it is unclear whether comparisons are made across the two species or these very different habitats. This sentence has been re-written in the revised manuscript: “Clutches of eggs laid at high-density sea turtle nesting beach aggregations such as green turtles on Raine Island, Australia, and olive ridley turtles (*Lepidochelys olivacea*) nesting in arribada aggregations in Costa Rica, typically experience much higher mortality of embryos (30-80% mortality) than clutches of eggs laid at low aggregation nesting aggregation beaches like Heron Island, Australia (0-10% mortality)[10,11,12,13].”

L72 Are there any studies that indicate an inability of young embryos to mount a heat-shock protein response? Please cite. A study is now cited: Tedeschi et al. (2015).

L72-79 This is a repetition of earlier statements and I suggest integrating it with previous mentions. These sentences have been shortened, but are still necessary to make the point that it is only the early stage embryos that are susceptible t mortality from these environmental stressors.

L79 I liked that the authors put forward a testable hypothesis in the abstract and I suggest doing the same here, in the introduction. I also recommend that the authors specifically introduce the conditions that are considered “optimal” for embryo development and how they are going to be manipulated in this study (i.e. replace “suboptimal”). These suggestions have been incorporated into the revised manuscript by changing the sentence early in the introduction “Typically, during the early stages of incubation, sea turtle embryos experience respiratory gases (oxygen and carbon dioxide) partial pressures close to those of the atmosphere above the sand [6,7] and temperatures in the range 28 - 30^o^C and these are considered to be optimal conditions for embryonic development.” And a sentence at the end of the introduction: “In the current study, through a set of controlled incubation experiments, we investigate if incubation conditions that vary significantly from optimal with respect to respiratory gases, elevated temperatures or a combination of these two factors increase the frequency of early embryo mortality in sea turtle embryos.”

Materials and Methods
L90-106 In this paragraph, mention the numbers of eggs that were collected. I was surprised to read in the results that there were several hundred eggs per clutch. If anything, this strengthens the paper. This information is including in the revised manuscript: “Loggerhead turtle (*Caretta cartetta*) eggs were collected (four clutches consisting of 162, 165, 166 and 157 eggs) during oviposition at Mon Repos beach (24.8059°S, 152.4416°E) between 4/12/2019 and 15/12/2019 and green turtle (*Chelonia mydas*) eggs were collected (four clutches consisting of 159, 132, 154 and 151 eggs) at Heron Island (23.4423°S, 151.9148°E) between 06/01/2019 and 19/01/2019.”

L97 I would avoid mentioning brand names. An insulated cooler or container. Done.

L95-106 I understand that there were some logistical challenges to getting the green turtle eggs into the lab. Presumably the careful transport of these embryos did not affect their development, but it would be good if the authors could justify this in a sentence or two. Were mortality rates generally comparable to clutches that were left undisturbed? Similar to those of the loggerhead turtles that did not experience the transport procedure? Or were they typical for what has been reported for this species in the past? This point has been addressed in the revised manuscript: “This temperature slows down the embryo developmental rate significantly so that the eggs do not undergo movement-induced mortality during transportation and the hatching rates of eggs transported in this way are similar to untreated eggs [17,18]”.

L136-139 I think the authors should provide a justification for why exactly these gas tensions were used. I understand that they span the conditions that turtles may experience in the wild, but why were exactly these combinations of O2/CO2 chosen? I.e. O2 is reduced in steps of 80%, 66% and 47% relative to normoxia, whereas CO2 is increased in steps of 4%, 7% and 11%. What’s the basis for choosing these ratios? Also, is the 21%/0% treatment really the best control, as wild turtles would probably never experience this in the sand. I’m not saying that there is something fundamentally wrong with these experimental conditions, but I think the authors need to justify specifically why they were chosen. This is now explained in a sentence added to the revised manuscript: “The ratio of oxygen to carbon dioxide was based on the ratios found in natural green turtle nests on Raine Island [13].”

L149-160 This section is a bit confusing as written and the authors should state more clearly what measurements were performed at which times? How do they know if the embryos grew (and were alive) before tracing the patch and comparing it to the previous measurement?
It seems that two different methods were used to assess whether an embryo was alive, tracing a white spot on the egg and staging embryos by dissection. Why this redundancy is there a reason to think that one method would yield different or unreliable results? And if so, why not use the more reliable of the two methods. And what exactly was assessed during staging of the embryos?
It’s also not clear to me why the embryos were only exposed to the gas mixtures for 7 days. And it sounds like some embryos were returned to their natural nests thereafter, but others were not. It’s not entirely clear to me how this helped addressing the research question. Please clarify. We have addressed these issues in the revised manuscript by re-writing this section of the methods: “We incubated all eggs for a further 5.5 days after initiation of the gas mixture exposure. Because we were only interested in investigating embryo death during the first week of incubation, we did not continue laboratory incubation of eggs past this point.

After 5.5 days of gas exposure we removed containers from incubators and examined the eggs. If the initial white-patch had not expanded in size, we assumed the embryo was dead and removed the egg, which we dissected and staged. If the white-patch had expanded we assumed the embryo was alive and traced the new white-patch boundary with a 2B pencil and photographed the egg from the side. An estimate of white-patch coverage as a percent of the entire egg surface was made using ImageJ (https://imagej.nih.gov/ij/). The increase in the area of the white patch from 36h to 5.5 days was used as an estimate of growth rate of the embryo. To confirm our assumption that embryos within eggs were alive we selected two eggs at random from each treatment (one from each of the two clutches) to be dissected and staged. Because both loggerhead and green turtles are endangered or threatened species, we did not kill all eggs to ascertain that they were living and to assess their size. We relocated the surviving eggs into artificial nests on Mon Repos Beach.”

L157-160 The authors mentioned earlier that one of the gas mixtures ran out and presumably this is why the last clutch of green turtle eggs was kept at ambient gas concentrations throughout, but instead temperature was manipulated, right? Yes that is correct.

L159-160 Include, how many eggs were sampled at each timepoint. This longer-term experiment has been removed from the revised manuscript.

Results
L178 The authors have not introduced the metric of developmental stages or how they were assessed. This should be described in the M&M section. We now reference the staging guide that was used.

L187-188 It’s still not clear to me exactly how these long-term experiments were performed. It sounds like all loggerhead eggs were returned to the wild after the 7 day exposure, but the green turtle eggs went back into the incubators. At this point gas tensions were ambient for all treatments (L159), but in figure 1 it says that data was pooled across gas treatments. And that we have one additional treatment that was only exposed to air throughout the experiment (because the gas ran out) and this treatment was kept at 34°C instead of 33°C (L138-140). I had to go back and forth several times to piece this information together and I’m still not sure whether this is all correct. I’m also unsure whether the comparisons that are being made are actually meaningful (see major comment about including a dedicated M&M section on experimental design). This long-term experiment has been removed from the revised manuscript.

L199-200 It’s ok to present only these data, but mention in a sentence whether the two metrics of embryo development agree with one-another or not. Perhaps the staging data could be made accessible in a supplement, to avoid cherry-picking one dataset over the other? This evidence is presented as a good correlation between the two methods.

L205-207 This sentence is confusing as written, please re-phrase. This sentence has been re-written in the revised manuscript: “The rate of growth of the white-patch decreased when the respiratory gases embryos were exposed to became increasing different from the optimal atmospheric condition of 21% oxygen, 0% carbon dioxide, for both loggerhead and green turtle embryos (Fig. 1).”

Tables
In general, I would avoid the vertical lines in tables for publication.
In the table captions I would mention the full species names, instead of just “loggerhead or green eggs”. We have included the species scientific name in the Table captions in the revised manuscript. We will rely on the copy editor;\’s advice about the format of the table lines.

Figure 1 I would avoid using the thick line in the 34°C treatment as is covers the actual datapoints. Chose a different linetype that is less occlusive. In my version, the R2 appeared as a box. If the data were pooled across gas treatments, I’d like to see some measure of variability included around the means (SD or SEM). The old Fig. 1 has been delete from the revised manuscript.

Were there any significant differences in survival between the embryos that had previously been exposed to the different gas tensions? If not mention this here as it justifies the pooling of these data. Otherwise report the result as it would be interesting to know whether hypoxia or hypocapnia during early development has an impact on long-term survival rates. The old Fig. 1 has been delete from the revised manuscript.

Figure 2 Since there were significant effects of gas tensions on embryo development these treatment should not be pooled. The presentation of these data will need to be changed. The figure will need to show the development for each gas tension individually and to highlight that there was a significant effect of temperature the authors could include the p-values for the main effects and the interaction term in the figure as well, which would replace Table 3.
The same considerations apply to Figure 3. I recommend the authors revisit the presentation of their data. I suggest presenting one figure per species, as no statistical comparisons are made between the two species. And each of these figures will need to show all combinations of experimental treatments as they were significantly different and there was a significant interaction between gas treatments and temperature, in each case. A more common way to express changes in development with temperature would be to calculate the Q10 values for each step. This is somewhat of a personal preference, but the authors should consider this type of analysis. The old figures 2 and 3 have been incorporated into a single new figure which was redraw as recommended.

Discussion
L217 the gases were not “sub-atmospheric” for CO2. This sentence has been re-written: “However, we found that as the respiratory gases deviated further from atmospheric air, the rate of embryo development decreased, so clearly there was a deleterious effect of prolonged exposure to respiratory gases that deviate from atmospheric conditions at this early stage of embryonic development.”

L222-226 I disagree with this statement as it neglects the possibility that there could be a number of adjacent clutches that are staggered in their developmental stage and therefore a single clutch of eggs may experience hypoxia and hypercapnia throughout development or for any fraction of this time. Thanks for pointing this possible circumstance out. We have revised the manuscript to take into account this possibility: “However, in nature, clutches are only exposed to self-generated low oxygen and high carbon dioxide partial pressures during the last 2 weeks of incubation. After this time, hatchlings escape the nest and oxygen and carbon dioxide partial pressures return to air conditions. So, even if a new clutch was laid next to a late maturing clutch, embryos in the new clutch most are likely only be exposed to adverse respiratory gases for a maximum of two weeks unless they also have multiple clutches with embryos at different developmental stages next to them as well.”

L227-230 I’m not sure this comparison is backed by the data and no statistical evidence is provided to support this. In addition, differences in the development of a “white patch”, a rather subjective marker, could simply be due to differences between egg size, egg-shell composition, etc. between the species. Based on the provided data it cannot be concluded that one species is more susceptible to the altered gas tensions than the other. This applies to the entire following paragraph from L227-245. As suggested by the reviewer we now exclude this section of the discussion from the revised manuscript.

L235-238 I think it’s great that the authors discuss critically the possibility that cooling the embryos of one species before the trials may have confounded the results. But I still think it would be good to dissipate these concerns earlier in the M&M section by providing a sound justification of this approach. I understand that field conditions are usually a compromise and if it was simply not possible to treat the eggs from both species in the same way that can be sated as well. Based on this discrepancy I would further discourage the authors from drawing any conclusions by comparing the development of the two species. The experimental design simply does not support these comparisons.

This particular comparison is now deleted from the revised manuscript, but the point about egg chilling on later embryonic growth is now included in the methods section.

L238-245 The authors focus on an O2 limitation as the probable cause for an impaired development. Also this cannot be concluded from the data, as O2 and CO2 were always altered in concert. I urge the authors to fundamentally revise the interpretation of their data and pay close attention to the limitations in their experimental design. I think the experimental design is appropriate to answer the main study question on whether embryonic development is affected by the conditions (gas and temperature) of nearby clutches on crowded beaches. I recommend the authors adhere closely to this question and avoid overinterpreting their data. The effects of O2 and CO2 on development of embryos can be discussed and some speculation is ok to suggest future experiments, but the effect of these gases cannot be disentangled unambiguously. Because of this problem the section of the discussion on oxygen limitation has been deleted from the manuscript.

L261-263 Again, based on the presented data I’d like to know whether early hypoxia or hypercapnia had an effect on late embryo mortality in ambient gas tensions. Did the authors test for such an effect? Whatever the finding, this would be interesting to report in the results.

The data and discussion on late embryo mortality has been deleted from the revised manuscript.

L269-272 I’m not convince that the slower development at higher temperatures (by itself) is an indication of cellular damage or morbidity. In fact, it could be an adaptive response of the embryos to prevent a further deterioration of the conditions surrounding the clutch and perhaps to allow for neighboring clutched to hatch first by delaying their own development. The only data that really suggests that the embryos may be pushed beyond their physiological tolerance is the survival data. I suggest the authors make this distinction more clearly. Thanks for pointing this possibility out. We have added a sentence in the revised manuscript to address this possibility; “Alternatively, slowing of development at high temperatures may be a tactic to prevent high temperature damage, by waiting until cooler temperatures return before recommencing development.”

L276 “clutches” Fixed.

L278 at 34°C long-term survival was affected. Again, here the results of the short-term and long-term experiments are intermixed. I recommend differentiating more clearly between effects on development (within the first 8.5 days) and long-term survival, which was affected by temperature. The long-term experiment data and discussion has been deleted from the revised manuscript.

L281-288 I like this last paragraph that interprets the major findings in an applied context and provides clear management strategies for turtle conservation. I suggest ending the abstract in a similar way. We thank the reviewer for this comment. We have added a sentence to the end of the abstract as suggested by the reviewer: “Any management strategies that can increase the spacing between nests or other strategies such as shading or irrigation that reduce sand temperature are likely to increase hatching success at high density nesting beaches.”
