# Supplementary material for: The effect of respiratory gases and incubation temperature on early stage embryonic development in sea turtles
Source: PLoS One. 2020 Dec 2;15(12):e0233580. doi: 10.1371/journal.pone.0233580 (PMC7710074; doi:10.1371/journal.pone.0233580)
Supplement: Attachment — Submitted filename: Response to reviewers October 2020.docx [file pone.0233580.s005.docx]

Response to reviewers’ comments.

Please thank the reviewers for their feedback and comments and suggestions. Below we detail our response to the reviewer’s comments. Reviewers’ comments are in *italics* and our responses are in regular font.

Reviewer 1:

General points:

*I am concerned that my main criticism of this manuscript has still not been fully addressed. The study aim is to assess the impact of changes in suboptimal gas mixtures and temperatures during early-embryonic development to ascertain whether this could explain high rates of early embryonic death at high-density nesting beaches. Data on stage of embryonic death has not been presented for sufficient comparison of differences between the treatments. Furthermore, in this revised version, there is no longer any substantial mortality data included. This makes it difficult to definitively conclude that any of the treatments applied caused higher rates of early-embryonic death and poorer hatching success in general. The authors did apparently open 2 eggs (or potentially 4?) from each treatment to assess stage of embryonic development at the end of the treatment but this data has not been reported in the manuscript.*

Response: The focus of the manuscript is early stage embryonic development, development that occurs within the first 7 days of the eggs being laid. The reason for this highly focused short period of incubation is that at high density nesting beaches, a high proportion of embryo mortality occurs during this period of development. There was virtually no early embryo death detected in any of our treatments during the first 7 days of incubation. However, for the few embryos that did die, we now report the stages that these embryos died at in the revised manuscript. A small sample of eggs were opened at the end of the 7 day treatment period, and all of these embryos were found to be alive. This is now reported in the revised manuscript: “All embryos were alive as indicated by a beating heart when dissected”.

*Given the above, if data on stage of embryonic death cannot be provided, the authors need to temper interpretation of results that temps above 34℃ are more harmful than non-optimal gas concentrations. There was minimal difference in the proportion of embryos that you assumed were still alive at 7 days across all treatments, including temperature treatments. Furthermore, the data you report on relative growth of the white-patch indicate that non-ideal gas mixtures (10%O2, 11%CO2) had a more pronounced negative impact on white-patch growth than temperatures of 34 degrees. There were also no major differences in early-embryo survival across your temperature treatments.*

Response: We agree with the reviewer’s comments, but our comments about temperatures above 34^o^C are based on previously published studies. We have adopted the reviewer’s suggestion and “tempered” our conclusion about high temperature death of early stage embryos, by replacing the would “likely” with “possible” in the revised manuscript: “Hence, high nest temperatures experienced early in incubation is a possible explanation for the large number of nests experiencing a high rate of early embryo death in high-density nesting aggregations.” And we have also tempered our conclusion later in the discussion: “At nesting beaches that experience high-density nesting, the close proximity of nests means that many newly laid clutches could experience high temperatures due to the metabolic heat production of nearby maturing clutches, and this might result in elevated rates of early embryo death, especially if temperatures exceed 34^o^C for long periods of time.”

*I am concerned by the response to my initial suggestion in the first round of review:*

*“Page 8, Lines 188-191: I assume you excavated and staged the unhatched eggs from the*

*clutches that were reburied in artificial nests as well. You should provide a summary of these*

*data also. Or explain why this was not done. If the primary concern of this study is early stage*

*death I think it is quite important to present the data on which stage of development these*

*embryos died at. This part of the study has now been deleted from the manuscript.”*

*This part of the study has not been deleted from the manuscript. The authors still state that eggs were reburied in artificial nests on Mon Repos Beach. Is there excavation data with data on stage of death for the eggs? Given the central concern of the study is early stage death, it is important that data on the stage that embryos died at is reported in as much detail as possible. Or provide explanation why this was not done.*

Response: If we had data on hatching success of eggs from each of our treatments, we certainly would have reported it, but we do not have this data. The reviewer has asked why we did not obtain this data. The reason is because of the logistics of obtaining such data. We had limited incubator space and time – we could not continue to incubate our eggs in incubators until they hatched – there was not enough incubator space. Our typical experiment group size for each clutch/treatment combination was 8-10 eggs. As the reviewer knows, such a small number of eggs incubated naturally at nest depth on a beach would not produce enough hatchlings to be able to dig themselves to the surface after they hatch, a group size of 25-30 hatchlings is needed. So, in our study we combined eggs from several clutch/treatments to form new clutches of 100/120 eggs that were incubated on the beach. Because within each of these combined clutches, we could not determine which hatchling came from which treatment, there was no point in evaluating the hatching success of these combined nests. The only reason we stated that eggs were buried to complete incubation on the beach was to assure readers that these eggs were not destroyed after our experimental manipulations. We have now added some further details to the methods section: “We relocated the surviving eggs into artificial nests consisting of 100 to 120 eggs pooled from the different treatments on Mon Repos Beach. Because we could not identify from which treatment hatchlings came from in these artificial nests, we could not assess the effect of treatment on hatching success, so this data is not reported.”

*Reviewer 2 brings up a similar point which could be answered if the hatching success and stage of embryonic death data is supplied for the eggs that were reburied in artificial nests at Mon Repos:*

*“L261-263 Again, based on the presented data I’d like to know whether early hypoxia or*

*hypercapnia had an effect on late embryo mortality in ambient gas tensions. Did the authors*

*test for such an effect? Whatever the finding, this would be interesting to report in the results.*

*The data and discussion on late embryo mortality has been deleted from the revised*

*manuscript.”*

Response: Unfortunately, we cannot make comment on this aspect because we do not know that the hatching success of eggs were. The only data we have on later embryo stage death was from the last two clutches of green turtle eggs incubated. We do not have this data for any of the loggerhead turtle experiments, or the first two clutches of green turtle eggs. So, as suggested by the reviewers in the first round of reviews, presenting this data added confusion to the main aim of assessing temperature and gas mixture effects on early stage death,. It is for this reason that we now do not report the data on later stage death of the last two clutches of green turtle eggs in the revised manuscript.

*This is the only major concern I still have with the manuscript; the rest below should be straightforward to fix.*

*Specific points:*

*Page 2, Line 35: Spacing either side of "/" needs to be fixed.* Response: done.

*Page 4, Line 73: "low aggregation nesting aggregation beaches" needs to be changed. Suggest "low density nesting beaches" or "low density nesting beach aggregations".* Response: This change has been made. Changed to “low density nesting beaches”.

*Page 4, Line 78: Change "with the majority of embryos die at an early stage" to "with the majority of embryos dying at an early stage of development".* Response: This change has been made as requested.

*Page 5, Lines 106-110: Maybe I've got this wrong. But it seems confusing to me that you ran out of one of the gas mixtures for the treatments for the green turtle experiment when the dates you've reported here suggest this experiment was undertaken first. I suggest double checking your dates for egg collections you have listed in the methods. Or revisit the wording you have used to explain the reason why this treatment was not included in the green turtle experiment.*  Response: Thanks for pointing this out. The loggerhead turtle eggs were collected in December 2018, not December 2019. This date correction has been made in the revised manuscript.

*Page 8, Lines 182-184: Here in the methods, the authors state that two eggs were randomly selected from each treatment (one from each of the "two clutches") to be opened and staged, but further down in the results they state that four eggs from each treatment (one per clutch) were opened and staged. Similarly, at the start of the methods you report four clutches per species being collected at the start of the methods. Furthermore, in the results you report sample numbers of eggs that were opened, 9 and 12 for green turtles and loggerheads respectively. These numbers don’t add up to me, please provide clarification.* Response: Thanks for pointing this inconsistency out. We have now corrected this mistake in the revised manuscript.

P*age 9, Line 213: You should present a summary of the data on developmental stage of these opened eggs. The data is not supplied in the raw data file either.* Response: This data is now reported in a new Table 3 in the revised manuscript.

*Page 10, Line 221: "all three factors, clutch, temperature and clutch influenced growth" needs to be changed to "all three factors, clutch, temperature and gas influenced growth"*. Response: This change has been made.

*Page 10, Line 226-227: "became increase different" needs to change to "became increasingly different"* Response: This change has been made.

*Page 15, Line 339: The journal title is abbreviated here whereas other journal titles in the list are not abbreviated. Change for consistency / journal style.* Response: This change has been made.

*Page 19, Line 435: Change "treatment in for green" to "treatment for green".* Response: This change has been made.
