# Supplementary material for: The effect of respiratory gases and incubation temperature on early stage embryonic development in sea turtles
Source: PLoS One. 2020 Dec 2;15(12):e0233580. doi: 10.1371/journal.pone.0233580 (PMC7710074; doi:10.1371/journal.pone.0233580)
Supplement: Attachment — Submitted filename: Reviewer comments.docx [file pone.0233580.s002.docx]

**Reviewer comments & suggested edits**

General thoughts:

The manuscript presents a sound study that answers an important biological question with real conservation consequences for threatened sea turtle populations. I congratulate the authors on designing and executing a valuable study to elucidate the causes of abnormally high early-stage embryonic death at high-density sea turtle nesting beaches.

My main criticism with this manuscript is that, for the two clutches where data was presented for stage of embryonic mortality, the temperature treatments were applied over the entire incubation period and therefore does not reflect a "short-term" early incubation effect of a nearby mature clutch incubating. In the clutches where high temperatures were only applied for the initial period of incubation the authors have not presented the data for stage of embryonic development at which the embryos died. I suggest that the authors either revise the manuscript to include the stage of death data for the unhatched eggs from clutches that were reburied in artificial nests. If these data do not exist, the authors need to address this limitation and I suggest they include a caveat in the discussion to highlight the limitation that embryonic death cannot be conclusively attributed to "short-term early-stage" high incubation temperature. This is the only major change I recommend making to the manuscript, the rest of the points I raise should be easy to address.

What about the impact of extended pre-ovipositional embryonic arrest on embryos ability to survive high nest temperatures and low oxygen partial pressure? At high-density nesting beaches, such as Raine Island and Ostional, it could be likely that there are higher rates of failed nesting attempts due to disturbance from other females. This would mean a higher proportion of females return to nest over subsequent days to attempt oviposition when compared with those nesting at low-density nesting beaches. Inherently, this would mean that embryos are maintained in embryonic arrest for longer periods of time. Previous experiments have found that sea turtle embryos have limited tolerance to remain in embryonic arrest (Rings et al. 2015 Phys. Bio. Zool. and Williamson et al. 2019 Sci. Rep.) and in one population of leatherback turtles longer interesting intervals result in higher proportions of early-stage embryonic mortality (Rafferty et al. 2011 PLoS One). If embryos are potentially compromised by extended arrest they may be more susceptible to higher incubation temperatures and lower oxygen availability when they do recommence development after the arrest. It would be good for the authors to include some acknowledgement of this possibility in the discussion.

Specific points:

Page 1, Line 14: Change to "high death rate" or "high mortality rate".

Page 1, Line 22: Change "sea turtle nest" to "sea turtle nests".

Pages 1 - 2, Lines 23 - 24: Change "did not influence embryo mortality of early stage embryos" to "did not influence the mortality rate of early stage embryos".

Page 2, Line 25: I am unsure what the current word count for the abstract is, but I suggest including a final sentence summarizing the importance and implications of the findings.

Page 2 Line 34: Delete "of" from "increases of lizard"

Page 2, Lines 36-37: Provide a reference for this sentence. Perhaps one of the Ackerman papers on the topic. I suggest also breaking this paragraph into two separate paragraphs. One on the information about developmental arrest and the other on the information about varying gas concentrations and temperature throughout incubation in the nest.

Page 2, Line 39: Change "stalls" to "arrests".

Page 2, Lines 39 - 40: Embryos do not break developmental arrest immediately after oviposition. The study that the authors cite for this sentence suggests that embryos break the arrest after at least 12 hours of exposure to higher oxygen partial pressures. Please change this sentence to reflect this difference.

Page 2, Line 42: Change "development arrest" to "developmental arrest".

Page 3, Line 53: Change "it released carbon dioxide" to "it releases carbon dioxide".

Page 3, Line 57: Change “aggregations such as” to “aggregation beaches, such as”.

Page 3, Line 58: Arribadas is plural in Spanish. Change "nesting in arribadas aggregations" to "nesting in arribadas" or "nesting in arribada aggregations"

Page 3, Line 68: Again, this paragraph is very long. Suggestion to split into two paragraphs after this sentence.

Page 3, Lines 70-72: Provide appropriate references for this section of the sentence. Possibly Nicki Mitchell et al papers on heat-shock proteins in sea turtle embryos e.g. Bentley et al. 2015 Mol. Ecol.; or Tedeschi et al 2015 or 2016.

Page 5, Line 111: Please provide more information on the type of containers used for the application of the gas treatments.

Page 5, Lines 113 - 114: Unless I have missed it, there is no explanation provided for the use of Raine Island sand during incubation. Please explain the reasoning for this.

Page 6, Line 131: Provide reference to the staging criteria that you staged embryos with. I assume Miller's 1985 staging guide?

Page 7, Line 159: Please provide clarification whether these eggs were maintained at their assigned temperature treatment or if all eggs were then incubated at a similar temperature.

Page 7, Line 160: Please explain a little more about what happened to these eggs. Were they opened and staged to assess viability? Were the eggs left to incubate until hatching in the incubators?

Page 8, Lines 188-191: I assume you excavated and staged the unhatched eggs from the clutches that were reburied in artificial nests as well. You should provide a summary of these data also. Or explain why this was not done. If the primary concern of this study is early stage death I think it is quite important to present the data on which stage of development these embryos died at.

Page 9, Line 198: This is an important finding and indicates that growth of the white spot is actively regulated by the embryo rather than passively by desiccation or other environmental influences.

Page 10, Line 228: Change "logger turtle" to "loggerhead turtle"

Page 10, Lines 235-237: It is important the authors have acknowledged this potential limitation in assessing the difference in growth rates between the two species given that they applied different methodologies to the eggs dependent on species.

Page 12, line 274: Change "early stage sea turtle embryos development rate" to "the development rate of early stage sea turtle embryos".

Page 19, Lines 426 – 427 and Page 20, Lines 430 - 431: Table 1 & 2 Captions are nonsensical to me. The experimental treatments cannot indicate survival in themselves. I suggest changing "turtle eggs indicating survival" to "turtle eggs and embryo survival" or something similar.
