# Supplementary material for: The effect of respiratory gases and incubation temperature on early stage embryonic development in sea turtles
Source: PLoS One. 2020 Dec 2;15(12):e0233580. doi: 10.1371/journal.pone.0233580 (PMC7710074; doi:10.1371/journal.pone.0233580)
Supplement: Attachment — Submitted filename: Reviewer comments.docx [file pone.0233580.s004.docx]

**Reviewer comments & suggested edits**

General points:

I am concerned that my main criticism of this manuscript has still not been fully addressed. The study aim is to assess the impact of changes in suboptimal gas mixtures and temperatures during early-embryonic development to ascertain whether this could explain high rates of early embryonic death at high-density nesting beaches. Data on stage of embryonic death has not been presented for sufficient comparison of differences between the treatments. Furthermore, in this revised version, there is no longer any substantial mortality data included. This makes it difficult to definitively conclude that any of the treatments applied caused higher rates of early-embryonic death and poorer hatching success in general. The authors did apparently open 2 eggs (or potentially 4?) from each treatment to assess stage of embryonic development at the end of the treatment but this data has not been reported in the manuscript.

Given the above, if data on stage of embryonic death cannot be provided, the authors need to temper interpretation of results that temps above 34℃ are more harmful than non-optimal gas concentrations. There was minimal difference in the proportion of embryos that you assumed were still alive at 7 days across all treatments, including temperature treatments. Furthermore, the data you report on relative growth of the white-patch indicate that non-ideal gas mixtures (10%O2, 11%CO2) had a more pronounced negative impact on white-patch growth than temperatures of 34 degrees. There were also no major differences in early-embryo survival across your temperature treatments.

I am concerned by the response to my initial suggestion in the first round of review:

“Page 8, Lines 188-191: I assume you excavated and staged the unhatched eggs from the

clutches that were reburied in artificial nests as well. You should provide a summary of these

data also. Or explain why this was not done. If the primary concern of this study is early stage

death I think it is quite important to present the data on which stage of development these

embryos died at. This part of the study has now been deleted from the manuscript.”

This part of the study has not been deleted from the manuscript. The authors still state that eggs were reburied in artificial nests on Mon Repos Beach. Is there excavation data with data on stage of death for the eggs? Given the central concern of the study is early stage death, it is important that data on the stage that embryos died at is reported in as much detail as possible. Or provide explanation why this was not done.

Reviewer 2 brings up a similar point which could be answered if the hatching success and stage of embryonic death data is supplied for the eggs that were reburied in artificial nests at Mon Repos:

“L261-263 Again, based on the presented data I’d like to know whether early hypoxia or

hypercapnia had an effect on late embryo mortality in ambient gas tensions. Did the authors

test for such an effect? Whatever the finding, this would be interesting to report in the results.

The data and discussion on late embryo mortality has been deleted from the revised

manuscript.”

This is the only major concern I still have with the manuscript; the rest below should be straightforward to fix.

Specific points:

Page 2, Line 35: Spacing either side of "/" needs to be fixed.

Page 4, Line 73: "low aggregation nesting aggregation beaches" needs to be changed. Suggest "low density nesting beaches" or "low density nesting beach aggregations".

Page 4, Line 78: Change "with the majority of embryos die at an early stage" to "with the majority of embryos dying at an early stage of development".

Page 5, Lines 106-110: Maybe I've got this wrong. But it seems confusing to me that you ran out of one of the gas mixtures for the treatments for the green turtle experiment when the dates you've reported here suggest this experiment was undertaken first. I suggest double checking your dates for egg collections you have listed in the methods. Or revisit the wording you have used to explain the reason why this treatment was not included in the green turtle experiment.

Page 8, Lines 182-184: Here in the methods, the authors state that two eggs were randomly selected from each treatment (one from each of the "two clutches") to be opened and staged, but further down in the results they state that four eggs from each treatment (one per clutch) were opened and staged. Similarly, at the start of the methods you report four clutches per species being collected at the start of the methods. Furthermore, in the results you report sample numbers of eggs that were opened, 9 and 12 for green turtles and loggerheads respectively. These numbers don’t add up to me, please provide clarification.

Page 9, Line 213: You should present a summary of the data on developmental stage of these opened eggs. The data is not supplied in the raw data file either.

Page 10, Line 221: "all three factors, clutch, temperature and clutch influenced growth" needs to be changed to "all three factors, clutch, temperature and gas influenced growth".

Page 10, Line 226-227: "became increase different" needs to change to "became increasingly different"

Page 15, Line 339: The journal title is abbreviated here whereas other journal titles in the list are not abbreviated. Change for consistency / journal style.

Page 19, Line 435: Change "treatment in for green" to "treatment for green".
